# Healthcare provider perspectives on barriers and facilitators to exercise promotion among breast cancer survivors: Differences across professions

**DOI:** 10.1007/s11764-026-02029-x

**Published:** 2026-05-08

**Authors:** Oliver W. A. Wilson, Emma Tian, Jacob D. Schneider, Eleanor M. Kerr, Ilse Rivera, Laura Q. Rogers, Wendy Demark-Wahnefried, Jinani Jayasekera

**Affiliations:** 1 Division of Intramural Research at the National Institute on Minority Health and Health Disparities, National Institutes of Health, Bethesda, MD, USA; 2 Ripple Effect Communications, Inc., Rockville, MD, USA; 3 Division of General Internal Medicine and Population Science, Department of Medicine, University of Alabama Birmingham, Birmingham, AL, USA; 4 Department of Nutrition Sciences, University of Alabama Birmingham, Birmingham, AL, USA

**Keywords:** Physical activity, Health Personnel, Communication, Health Promotion

## Abstract

**Purpose:**

The extent to which exercise promotion (discussion and/or referral) barriers and facilitators in the care of breast cancer survivors differ across healthcare professions is currently unclear. This study aimed to describe differences in barriers and facilitators based on profession.

**Methods:**

Healthcare providers (oncologists/primary care physicians, cancer exercise-related professionals, allied healthcare providers) who had cared for breast cancer survivors aged ≥ 35-years in the past year participated in an online survey regarding cancer survivor exercise promotion barriers and facilitators. Prevalence ratios (PR) comparing differences in agreement for barriers and facilitators across professions were computed using Modified Poisson regression models.

**Results:**

Data were collected from 177 providers, including oncologists / primary care physicians (n = 68, 38.4%), exercise-related professionals (n = 67, 37.9%), and allied healthcare providers (n = 42, 23.7%). Differences were found across professions. For instance, compared with exercise-related professionals, oncologists / primary care physicians had a higher prevalence of agreement that limited time to discuss exercise (PR = 1.48, 95%CI:1.12–1.94), exercise being medically unsafe for patients (PR = 1.62, 95%CI:1.06–2.49), and not being convinced of the exercise and cancer outcomes literature (PR = 2.00, 95%CI:1.03–3.91) were barriers to exercise promotion.

**Conclusions:**

Exercise promotion barriers and facilitators appear to differ across professions. Exercise-related professionals appear to be less constrained by a lack of time and concerns about the safety of exercise, while oncologists appear unconvinced of exercise and cancer outcome evidence.

**Implications for Cancer Survivors:**

Exercise promotion efforts should consider the available time and competing responsibilities of each survivorship care team member, and better disseminate information regarding the safety and benefits of exercise to mitigate the risk of healthcare providers being unaware.

## Introduction

Advances in breast cancer screening and treatment have contributed to a reduction in breast cancer mortality by 58% between 1975 and 2019 in the United States (U.S.) [1]. Improved survival rates mean that an increasing number of breast cancer survivors are living with the side effects of past or continuing treatment including worse cardiovascular health and poor quality-of-life [2–5] A growing body of evidence shows that exercise could help reduce treatment side effects, improve cardiovascular health, quality-of-life, and survival among breast cancer survivors [6–9].

Despite the benefits of exercise and clinical guidelines for exercise endorsed by the American Society of Clinical Oncology (ASCO), American Cancer Society (ACS), and American College of Sports Medicine (ACSM) [6, 10, 11], 86.7% of breast cancer survivors do not participate in the recommended levels of exercise for health (≥ 150 min/week of aerobic exercise and ≥ 2 days/week of muscle-strengthening exercise) [12], and exercise participation is worse among lower socioeconomic status and rural breast cancer survivors [12, 13]. Healthcare providers are well-positioned to promote exercise among cancer survivors [14], especially as communicating information about exercise to breast cancer survivors has been shown to increase survivors’ exercise participation [15].

The ACSM and American Medical Association (AMA) have advocated for exercise promotion for all adults within healthcare settings since 2007 [16–18]. By contrast, the Moving Through Cancer Initiative, which aims to make exercise standard in oncology practice, was established relatively recently in 2019 [19]. Exercise is currently a component of ASCO cancer treatment and survivorship care plans [20] and, as of 2024, documentation of exercise recommendations by medical oncologists has been required for breast cancer center national accreditation in the U.S. [21]. The emphasis on exercise promotion for breast cancer survivors within clinical settings appears warranted, though approximately half of U.S. breast cancer survivors report having not discussed exercise with a healthcare provider (45.6%) [22] or received exercise advice from a healthcare provider (47.7%) [23].

A range of barriers may contribute to low rates of exercise promotion for cancer survivors in clinical settings. The most common barrier is a lack of time [14, 24–28], followed by a lack knowledge and/or confidence [25, 28–30], and support structures (e.g., limited referral options) [27, 30, 31]. These barriers highlight the importance of sharing the responsibility of exercise promotion across the survivorship care team, and the value in expanding the team to include an exercise specialist. Indeed, the inclusion of an exercise specialist as a part of the clinical team, as well as practitioner education sessions and patient handouts, are among the top reported facilitators that could help healthcare providers overcome barriers to exercise promotion for cancer survivors [32, 33]. Understanding the unique barriers and potential facilitators of different professions that comprise survivorship care teams is important to inform the design of feasible and acceptable interventions. However, at present, there is limited evidence describing how barriers and facilitators differ across members of the healthcare team. Therefore, the overarching aim of this study was to examine differences in barriers and facilitators based on healthcare profession.

## Methods

Data were collected as a part of an exploratory study examining the perspectives of healthcare providers on a prototype clinical decision tool designed to facilitate exercise discussions between providers and breast cancer survivors [34]. The National Institutes of Health (NIH) Institutional Review Board approved this study, which was considered exempt research based on the use of de-identified data.

### Survey development

Items were adapted from pre-existing instruments. A detailed description is available in Supplementary Materials and Methods S1. Cognitive interviews were conducted with 10 providers to assess their understanding of the survey questions/items and solicit input on any questions or concepts they considered missing or needing revision. Feedback was used to further refine the survey questions/items.

### Participants

We aimed to recruit ≥ 150 healthcare providers using convenience and snowball sampling. Healthcare providers recruited included breast oncologists/primary care physicians, exercise-related professionals (clinical exercise physiologists, cancer exercise trainers/specialists, and/or occupational/physical therapists), and allied healthcare providers (nurse practitioner, nurse, physician associate, patient navigator or social worker) who had treated or provided care to a breast cancer survivor (i.e., women diagnosed with breast cancer) aged ≥ 35-years in the past 12 months (June 2023-June 2024). Participants were recruited by an external contractor via professional organizations (e.g., ASCO) and publicly available information. To enhance data quality, eligibility screening questions were used to confirm respondents’ professional roles prior to survey access. Responses were also reviewed for indicators of low-quality or potentially ineligible participation, including incomplete surveys, implausible response patterns, and excessively short completion times. All participants provided informed consent and received $125-$150 for each cognitive interview (i.e., for survey development described above), and $60–75 for completing the online survey.

### Measures

#### Healthcare provider and practice characteristics

Providers self-reported their age (years), length of professional experience (years), healthcare profession(s) [35], race [36] and/or ethnicity [36], exercise training/education (formal and/or continuing), and exercise participation [37] using validated and established instruments (see Supplemental Materials and Methods S1). Information on the type of practice (e.g., community) [33] and location (e.g. rural) [38] was also collected. Providers, except for exercise-related professionals, were asked if they had the ability to provide a referral to an exercise-related professional within and/or outside of their healthcare network. Providers were also asked to report the percentage (0–100%) of breast cancer survivors under their care they had offered ‘personalized’ (i.e., individualized) exercise advice.

### Exercise promotion barriers and facilitators

Exercise promotion (i.e., discussion and/or referral) barriers and facilitators, specifically in relation to breast cancer survivors, were measured using five-point Likert scales ranging from strongly disagree (1) to strongly agree (5) with items adapted from the ‘Clinicians Perspectives on Exercise in Patients with Cancer’ (CliPEC) questionnaire [35]. Providers were asked to rate their agreement with 15 barriers and 12 facilitators. Barriers regarding the provision of an exercise referral were only rated by oncologists/primary care physicians and allied healthcare providers.

### Analyses

Descriptive statistics were computed using Stata (version 18.0) and Microsoft Excel. A ‘missing’ category was created for missing values for participant characteristics. Barriers and facilitators were described by provider type (oncologists/primary care physicians vs. exercise-related professionals vs. allied healthcare providers) by calculating percent agreement (i.e., those who somewhat agreed or strongly agreed) and Wilson 95% confidence intervals (CIs) for each item. Unadjusted differences in agreement across exercise-related professionals and oncologists/primary care physicians and allied healthcare providers were examined using chi-square tests. Adjusted differences in agreement across the professions were examined using Modified Poisson regression models (complete case analysis) to compute prevalence ratios (PR) adjusting for exercise promotion education (any formal and/or continuing exercise promotion education vs. nothing) and provider physical activity level (meeting guidelines vs. not meeting guidelines). Exercise-related professionals were chosen as the reference group, as they had the highest level of formal and/or continuing education in exercise promotion. Statistical significance was defined as p < 0.05.

## Results

### Healthcare provider characteristics

Healthcare provider characteristics are reported in Table 1. Our analytic sample comprised 177 providers. The median (interquartile range [IQR]) age of the overall sample was 43 (34–53) years, and most providers were women (67.2%) and non-Hispanic White (60.2%). Most providers were located in the West (32.8%), followed by the South (30.5%), North-east (25.4%) and Midwest (10.7%) regions [39]. The median professional experience overall was 8 (IQR = 5–13) years, and approximately half reported receiving formal (49.2%) or continuing (51.4%) education regarding exercise. More exercise-related professionals reported receiving formal and/or continuing education (92.5%) regarding exercise promotion compared to oncologists/primary care physicians (54.4%) and allied healthcare providers (40.5%). Most participants reported meeting exercise guidelines (67.8%) and practiced in urban/suburban settings (91.5%). Most oncologists/primary care physicians (91.2%) and allied healthcare providers (88.1%) had the ability to refer patients to an exercise program. Nearly all exercise-related professionals reported offering breast cancer survivors individualized exercise advice (Median [Md] = 97%; IQR = 71–100) compared to over half of oncologists/primary care physicians (Md = 65%; IQR = 50–73), and less than half of allied healthcare providers (Md = 41%; IQR = 10–65).

### Barriers

**Fig. 1:**
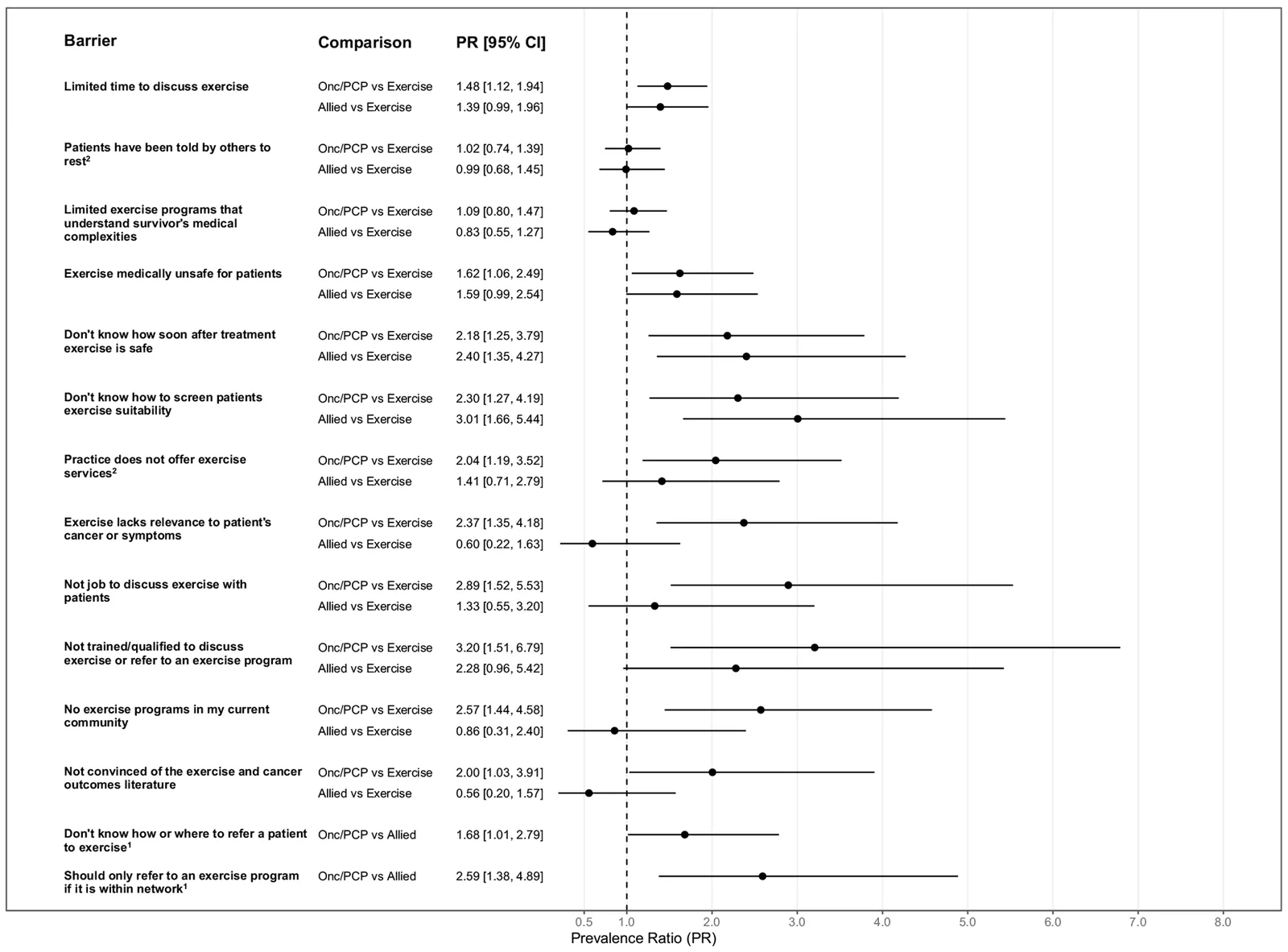
Adjusted differences in barriers to exercise promotion among breast cancer survivors across professions. Notes. CI = Confidence Interval; Onc/PCP = Oncologists/Primary care physicians; Allied healthcare professionals = Allied; Exercise = Exercise-related professionals; Modified Poisson regression models were adjusted for exercise promotion education (any formal and/or continuing exercise promotion education vs. nothing) and provider physical activity level (meeting guidelines vs. not meeting guidelines); Unless otherwise noted, estimates are based on N=175 respondents; ^1^Participants who were exclusively exercise-related professionals did not respond to this question: estimates compare Onc/PCP vs. Allied only (N=110); ^2^N=174 due to a missing item response

Descriptive statistics regarding barriers are reported in Table 2. Unadjusted differences in barriers across professions are reported in Table 2 and Supplemental Table 1, while adjusted are reported in Fig. 1. Overall, the three barriers with the highest agreement were limited time, patients being told by others to rest, and limited exercise programs that understand survivor’s medical complexities (Table 2). Being unconvinced of the exercise and cancer outcomes literature, an absence of exercise programs within their current community, and being untrained/unqualified to discuss exercise or refer to an exercise program were the barriers with the lowest agreement across all professions (Table 2). However, it is still worth noting that nearly 40% of oncologists/primary care physicians were not convinced about the exercise and cancer outcomes literature, and close to 60% of oncologists/primary care physicians and allied healthcare provider agreed exercise medically unsafe for some of the patients they treat (Table 2).

In adjusted analyses, differences across professions were found for several barriers to exercise promotion (Fig. 1). For instance, compared with exercise-related professionals, oncologists/primary care physicians had a two-fold higher prevalence of agreeing that being unconvinced of the exercise and cancer outcomes literature (PR = 2.00, 95%CI:1.03–3.91) was a barrier. Similarly, oncologists/primary care physicians were more likely to agree that exercise being medically unsafe for patients (PR = 1.62, 95%CI:1.06–2.49); exercise lacking relevance to patient’s cancer or symptoms (PR = 2.37, 95CI%:1.35–4.18); not being trained/qualified to discuss exercise or refer to an exercise program (PR = 3.20, 95%CI:1.51–6.79); no present community exercise programs (PR = 2.57, 95%CI:1.44–4.58); and limited time to discuss exercise (PR = 1.48, 95%CI:1.12–1.94) were barriers when compared with exercise-related professionals. Compared to exercise-related professionals, both oncologists/primary care physicians and allied healthcare providers had more than 25% higher prevalence of agreement with not knowing how soon after treatment exercise is safe and not knowing how to screen patient exercise suitability as being barriers to exercise promotion.

Only oncologists/primary care physicians and allied healthcare providers were presented with barrier items pertaining to referrals. Compared to allied healthcare providers, the prevalence of agreement among oncologists/primary care physicians was greater for not having knowledge on how or where to refer a patient to exercise (PR = 1.68, 95%CI:1.01–2.79) and should only refer to an in-network exercise program (PR = 2.59, 95%CI:1.38–4.89).

### Facilitators

**Fig. 2:**
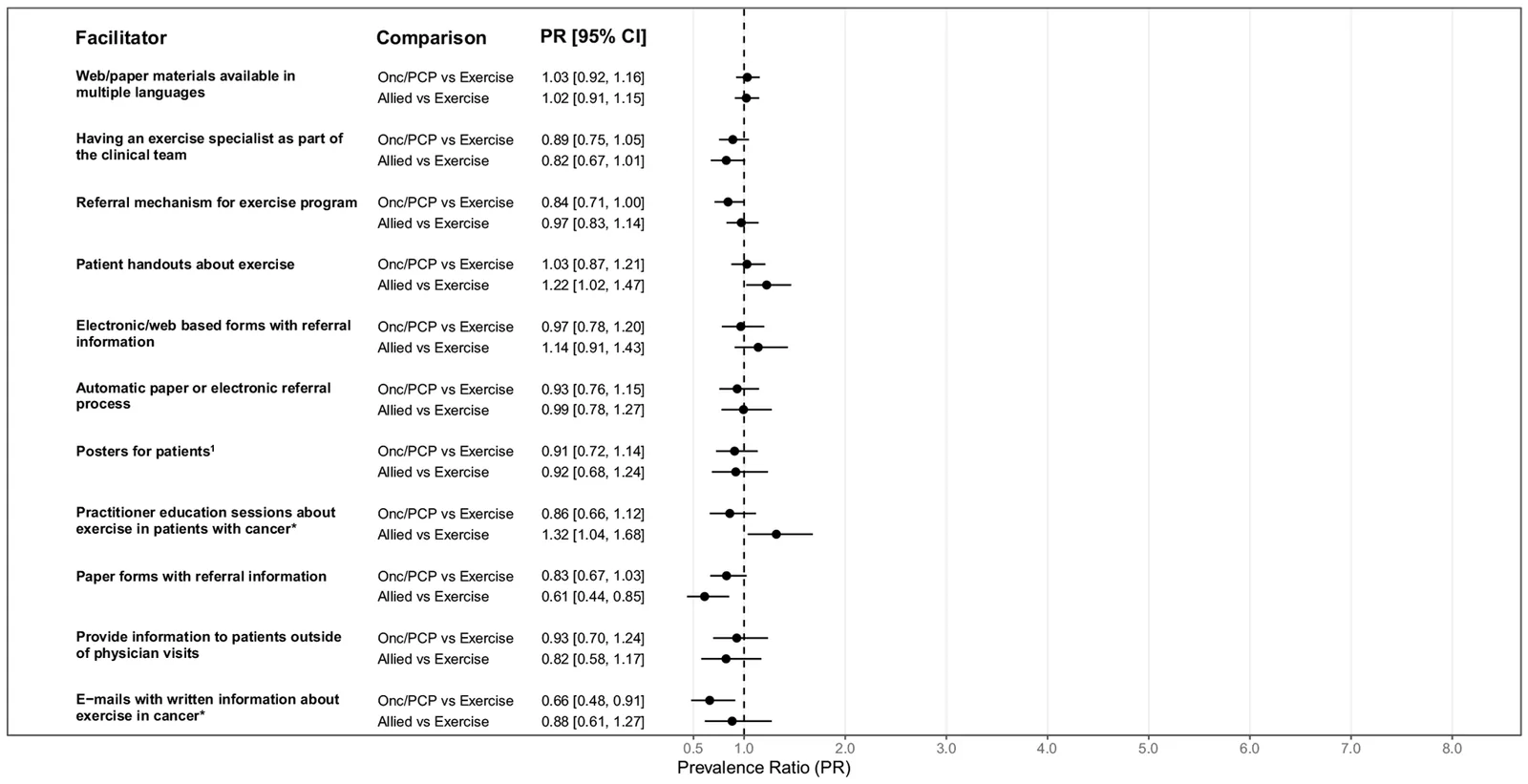
Adjusted differences in facilitators to exercise promotion among breast cancer survivors across professions. Notes. CI = Confidence Interval; Onc/PCP = Oncologists/Primary care physicians; Allied healthcare professionals = Allied; Exercise = Exercise-related professionals; *Indications, guidelines, referral process, safety information, etc.; Modified Poisson regression models (complete case analysis) were adjusted for exercise promotion education (any formal and/or continuing exercise promotion education vs. nothing) and provider physical activity level (meeting guidelines vs. not meeting guidelines); Unless otherwise noted, estimates are based on N=175 respondents; ^1^N=174 due to a missing item response

Descriptive statistics regarding facilitators are displayed in Table 2. Unadjusted differences in facilitators across professions are reported in Table 2 and Supplemental Table 2, while adjusted differences are reported in Fig. 2. Overall, five facilitators had ≥ 80% agreement that they would be helpful: 1) multilingual materials; 2) an exercise specialist being part of the clinical team; 3) an exercise program referral mechanism; 4) patient handouts about exercise; and 5) electronic/web-based forms with referral information. Overall, and across professions, a small proportion agreed that providing patients with exercise information outside of physician visits would be helpful (overall: 62.2%; 95% CI: 54.8–69.0%). There was also low agreement in support of emails with written information about exercise for cancer survivors among healthcare providers overall (overall: 58.8%; 95% CI: 51.4–65.8%), and particularly among oncologists/primary care physicians (47.1%; 95% CI: 35.7–58.8%).

In adjusted analyses, there were some differences in facilitators across professions. Compared to exercise-related professionals, the prevalence of agreement for the following facilitators was greater among allied healthcare providers: patient handouts about exercise (PR = 1.22, 95%CI:1.02–1.47) and practitioner education sessions about exercise in patients with cancer (PR = 1.32, 95%CI:1.04–1.68). Compared to exercise-related professionals, the prevalence of agreement for paper forms with referral information was lower among allied healthcare providers (PR = 0.61, 95%CI:0.44–0.85) and e-mails with written information about exercise in cancer was lower for oncologists/primary care providers (PR = 0.66, 95%CI:0.48–0.91).

## Discussion

Consistent with previous research, lack of time and knowledge were reportedly the greatest barriers [14, 24–31], while inclusion of an exercise specialist as a part of the clinical team and patient handouts were among the greatest facilitators [32, 33]. In alignment with prior research [27, 40], our findings also suggest that the majority of healthcare providers agree that discussing exercise with survivors is their responsibility.

Our adjusted analyses revealed that barriers and facilitators differed across professions. For example, oncologists/primary care physicians and allied healthcare providers perceived a lack of knowledge of how to screen for exercise suitability and how soon after treatment it is safe to exercise as greater barriers compared to exercise-related professionals. Similarly, practitioner education sessions were perceived as a greater facilitator for allied healthcare providers than exercise-related professionals. Findings suggesting some healthcare providers may have limited awareness regarding the safety and benefits of exercise are also worth noting.

Clinical guidelines (e.g., ACSM, ASCO, the National Comprehensive Cancer Network) state that exercise is safe [6, 10, 41, 42]. In addition to guidelines, cancer-specific medical triage systems (i.e., frameworks and/or algorithms) offer guidance to clinicians for exercise recommendations and referrals. Examples of these systems include the CaReR, (Cancer Rehabilitation to Recreation) Framework [43], CREST (Cancer Rehabilitation and Exercise Screening Tool) [44], the CORE (cardio-oncology rehabilitation) framework/algorithm [45–48], the Exercise in Cancer Evaluation and Decision Support [EXCEEDS] algorithm [49, 50], and PERCS (Personalized Exercise Rehabilitation in Cancer Survivorship) triage and referral system [51]. Future research could help develop and evaluate strategies to increase clinicians’ awareness, engagement, and uptake of these systems in clinical practice.

Recent meta-analytic and simulation modeling studies have reported the absolute benefits of different types and amounts of exercise for breast cancer survivors [8, 52]. The meta-analysis reported that meeting aerobic exercise guidelines (≥ 150-min/wk of moderate-intensity) was associated with an 8-percentage-point reduction in 5-year all-cause mortality compared to no/minimal aerobic exercise (< 45-min/wk of moderate-intensity) [8]. Similarly, the modeling study found increasing aerobic activity from < 30-min/wk to 150- < 300-min/wk was associated with an increase in 10-year all-cause survival by 5.3 percentage points among post-menopausal women diagnosed with stages I-III breast cancer [52]. These findings are comparable to the Colon Health and Life-Long Exercise Change (CHALLENGE) trial which showed that structured exercise among colon cancer survivors resulted in an absolute 7.1-percentage-point reduction in 8-year all-cause mortality (95% CI: 1.8–12.3) [53].

Most oncologists/primary care physicians and allied healthcare providers reported that they had the ability to refer breast cancer survivors to an exercise program. Yet, most also indicated that there were limited exercise programs that understand the medical complexities of survivors. This aligns with previous research that highlighted the lack of appropriate exercise programs to refer cancer survivors to, in part due to the absence of systematic integration of exercise oncology programs in the U.S. [54]. As a result, U.S. healthcare providers may be constrained to recommending community-based exercise programs which typically impose out-of-pocket expenses, or referring patients to exercise physiologists whose services are currently ineligible for health insurance coverage [55].

The current dearth of comprehensive coverage may explain why clinical exercise physiologists and cancer exercise specialists are not currently listed as a part of cancer care teams in the U.S [56, 57]. At present, cancer care teams in the U.S. include occupational and physical therapists, as well as dietitians who are identified as having expertise in exercise [57]. Despite the lack of coverage, our findings indicate that there is a desire for exercise specialists to be a part of care teams, and that time may be less of a barrier for exercise-related professionals (i.e., clinical exercise physiologists, cancer exercise specialists, occupational/physical therapists). By contrast, consistent with previous studies, limited time was the greatest barrier to exercise promotion among other healthcare providers [14, 24–27]. This could be explained by the differing amount of time healthcare providers are able to spend with each survivor in general, as well as time to talk about exercise specifically. For example, oncologists and primary care physicians in the U.S. spend an average of ~ 20 minutes with each patient and must address numerous topics in addition to exercise [58, 59]. However, recent changes made by Centers for Medicare and Medicaid Services that allow clinicians to bill for 15 minute physical activity assessments may begin to address structural issues concerning insurance and reimbursement and facilitate incorporation of exercise professionals into the reimbursable care pathway [60].

The lack of time also may explain why oncologists/primary care physicians and allied healthcare providers highly rated patient handouts about exercise and electronic forms with referral information compared to other facilitators. Each of these resources may reduce visit length and are consistent approaches that have been developed to support offering exercise prescriptions to or triage to services for cancer survivors. One example is the ACSM’s Moving Through Cancer (MTC) initiative and cancer exercise guidelines which encourage oncologists to “assess, advise, and refer”[6, 11, 61].

Various cancer-specific medical triage systems have also been developed to guide exercise referrals [43–51]. However, neither the MTC initiative or any of the triage systems support providers to offer individualized advice as recommended by cancer exercise guidelines [6–8]. Recently published evidence indicated that more than 80% of healthcare providers agreed that a tool that supports providers to offer breast cancer survivors with individualized exercise recommendations would be useful and used regularly [34].

Knowledge related barriers were higher among allied healthcare providers, who also rated practitioner education sessions and having a mechanism to refer patients highly as facilitators. A lack of knowledge on the part of allied healthcare providers is consistent with prior research [33]. These findings also align with less than half of allied healthcare providers reporting formal and/or continuing education in exercise promotion, and reinforce that more comprehensive education regarding exercise promotion is needed [33]. Equipping allied healthcare providers to promote physical activity is important, as informal physical activity guidance and support may fall on healthcare professionals beyond oncologists and primary care physicians [62].

Several cancer-specific exercise-related certifications are now available. The ACSM-ACS Cancer Exercise Specialist course is common among those with an exercise background [63]. The Klose Certified Lymphedema Training [64] and Psychological Oncology Rehabilitation Institute Breast Cancer Rehabilitation [65] qualifications are popular among physical and occupational therapists. Other common qualifications included Klose’s Strength after Breast Cancer [66], Cancer Exercise Training Institute courses [67], and the American Council on Exercise Cancer Exercise Specialist course [68]. However, these courses may present a steep learning curve for individuals without prior physical activity education, which is largely absent from non-exercise- or rehabilitation-focused healthcare provider curricula in the U.S. [69–71], despite calls to include basic physical activity education in the training of all healthcare providers [72]. While elevating the exercise knowledge of the entire cancer care team may prove challenging, the recent identification of core competencies is a helpful step in integrating exercise into standard oncology care [73].

There was consensus across professions that exercise information should not be provided to survivors outside clinical visits. This may be explained by the need to clarify that survivors are safe to engage in exercise, which the MTC initiative and EXCEEDS algorithm help to establish [6, 11, 49, 50, 61, 74]. Moreover, many breast cancer survivors in the U.S. seek out health-related information online [75, 76], including information about exercise [77], to meet their specific informational needs [78]. Online health-related information seeking by breast cancer survivors is influenced by factors such as age, education, income, and health literacy [79]. Though survivors tend to prefer established sources, such as the ACS or National Cancer Institute [76, 79], the readability of online information offered by such sources significantly exceeds the recommended level [80]. The tendency for breast cancer survivors to seek information online, and the poor readability of such information, may also explain why healthcare providers prefer that exercise information is provided to survivors in clinical visits.

Multilingual materials were rated the top facilitator across professions. Such materials may help to better meet the informational needs of breast cancer survivors. In particular, survivors with lower health literacy and non-English speaking survivors who have higher unmet informational needs [81, 82]. However, it is important that such information is high quality and provided at an appropriate reading level [83] given health literacy is associated with physical activity [84].

A notable strength of this study was the recruitment of a racially diverse sample of healthcare providers. However, it also had several limitations, namely the modest sample recruited solely from the U.S. Though our sample allowed for describing differences between broad types of healthcare providers, it was too small to compare differences in barriers and facilitators between specific professions, level of professional experience, or location (i.e., urban vs. rural). Future researchers should recruit larger samples to allow comparisons between different professions (e.g., clinical exercise physiologists vs. physical therapists) and contexts as professional responsibilities, including the responsibility to promote exercise, may vary between professions and healthcare systems.

## Conclusions

Our results offer insights that have practical implications for physical activity promotion for breast cancer in clinical settings. Many healthcare providers feel obligated to discuss exercise with survivors, but are constrained by a lack of time, knowledge, and a lack of exercise programs that understand the medical complexities of survivors. Findings suggest a demand for continuing physical activity education among allied healthcare providers, and reinforce the need for formal education in physical activity promotion for all healthcare providers [72]. In addition, our findings indicate that issues regarding limited awareness concerning the safety and benefits of exercise may need to be addressed for some providers. The delivery of individualized exercise recommendations for breast cancer by healthcare providers will need to consider existing demands on provider time, raise provider awareness of the safety and benefits of exercise, and facilitate the delivery of individualized information in multiple languages.

## Supplementary Material

Supplement

The online version contains supplementary material available at https://doi.org/10.1007/s11764-026-02029-x.

## Figures and Tables

**Table 1:** Healthcare provider characteristics

	Overall (n = 177)	Oncologists/Primary care physicians (n = 68)	Exercise-related professionals (n = 67)	Allied healthcare providers (n = 42)
**Age (years)**, Md (IQR)	43 (34–53)	48 (40–55)	42 (33–54)	40 (32–51)
**Professional experience (years),** Md (IQR)	8 (5–13)	8 (5–11)	7 (4–14)	9 (6–17)
**Gender**, n (%)				
Men	57 (32.2%)	32 (47.1%)	17 (25.4%)	8 (19.0%)
Women	119 (67.2%)	36 (52.9%)	49 (73.1%)	34 (81.0%)
Missing	1 (0.6%)	0	1 (1.5%)	0
**Race and/or ethnicity**, n (%)				
Asian	15 (8.5%)	9 (13.2%)	4 (6.0%)	2 (4.8%)
American Indian/Alaska Native	5 (2.8%)	2 (2.9%)	2 (3.0%)	1 (2.4%)
Non-Hispanic Black or African American	35 (19.8%)	21 (30.9%)	11 (16.4%)	3 (7.1%)
Hispanic or Latino	2 (1.1%)	0	1 (1.5%)	1 (2.4%)
Native Hawaiian/Pacific Islander	2 (1.1%)	1 (1.5%)	0	1 (2.4%)
Non-Hispanic White	107 (60.5%)	32 (47.1%)	44 (65.7%)	31 (73.8%)
Mixed/Other	11 (6.2%)	3 (4.4%)	5 (7.5%)	3 (7.1%)
**Education regarding exercise promotion**, n (%)				
Formal	87 (49.2%)	30 (44.1%)	46 (68.7%)	11 (26.2%)
Continuing	91 (51.4%)	28 (41.2%)	51 (76.1%)	12 (28.6%)
Formal and/or Continuing	116 (65.5%)	37 (54.4%)	62 (92.5%)	17 (40.5%)
**Exercise behavior**, n (%)				
Meeting guidelines^1^	120 (67.8%)	54 (79.4%)	42 (62.7%)	24 (57.1%)
**Rural/urban status**, n (%)				
Urban/suburban	162 (91.5%)	67 (98.5%)	59 (88.1%)	36 (85.7%)
Rural	15 (8.5%)	1 (1.5%)	8 (11.9%)	6 (14.3%)
**Ability to refer patients to an exercise program,**^2^ n (%)				
No	10 (9.1%)	6 (8.8%)		4 (9.5%)
Yes (within and/or outside network)	99 (90.0%)	62 (91.2%)		37 (88.1%)
Missing	18 (1.0%)	0		1 (2.4%)
**Proportion of breast cancer survivors offered ‘personalized’ (i.e., individualized) exercise advice (%),** Md (IQR)	59 (50–72)	65 (50–73)	97 (71–100)	41 (10–65)

Notes.

1 Guidelines = ≥ 150 moderate-intensity mins/week of aerobic exercise and ≥ 2 days/week of muscle-strengthening exercise;

2 Participants who were exclusively exercise-related professionals did not respond to this question

**Table 2:** Percentage agreement on barriers and facilitators for exercise promotion among breast cancer survivors across professions

	Overall (N=177)			Exercise-related professionals (N=67)			Oncologists/Primary care physicians (N=68)			Allied healthcare professionals (N=42)		
	%	95% CI		%	95% CI		%	95% CI		%	95% CI	
		Lower	Upper		Lower	Upper		Lower	Upper		Lower	Upper
**BARRIERS**												
Limited time to discuss exercise	66.7%	59.4%	73.2%	53.7%	41.9%	65.1%	77.9%	66.7%	86.2%	69.1%	54.0%	80.9%
Patients told to rest by others^2^	59.7%	52.3%	66.6%	59.1%	47.1%	70.1%	61.8%	49.9%	72.4%	57.1%	42.2%	70.9%
Limited exercise programs that understand survivor’s medical complexities	55.9%	48.6%	63.0%	58.2%	46.3%	69.3%	58.8%	47.0%	69.7%	47.6%	33.4%	62.3%
Exercise medically unsafe for patients	48.6%	41.3%	54.9%	32.8%	22.8%	44.7%	57.4%	45.5%	68.4%	59.5%	44.5%	73.0%
Don’t know how soon after treatment exercise is safe	42.9%	35.9%	50.3%	20.9%	12.9%	32.1%	52.9%	41.2%	64.3%	61.9%	46.8%	75.0%
Don’t know how to screen patients for exercise suitability	38.4%	31.6%	45.8%	17.9%	10.6%	28.8%	44.1%	33.0%	55.9%	61.9%	46.8%	75.0%
Practice does not offer exercise services^3^	37.5%	30.7%	44.9%	23.9%	15.3%	35.3%	51.5%	39.8%	63.0%	36.6%	23.6%	51.9%
Exercise lacks relevance to patient’s cancer/symptoms	33.9%	27.3%	41.2%	22.4%	14.1%	33.7%	57.4%	45.5%	68.4%	14.3%	6.7%	27.8%
Not job to discuss exercise with patients	29.4%	23.2%	36.5%	17.9%	10.60%	28.8%	47.1%	35.7%	58.8	19.1%	10.0%	33.3%
Not trained/qualified to discuss exercise or refer to an exercise program	28.3%	22.1%	35.3%	11.9%	6.2%	21.8%	44.1%	33.0%	55.9%	28.6%	17.2%	43.6%
No exercise programs in my current community	27.1%	21.1%	34.1%	19.4%	11.7%	30.4%	44.1%	33.0%	55.9%	11.9%	5.2%	25.0%
Not convinced of the exercise and cancer outcomes literature	25.4%	19.6%	32.3%	19.4%	11.7%	30.4%	39.7%	28.9%	51.6%	11.9%	5.2%	25.0%
Don’t know how or where to refer a patient to exercise^1^	44.6%	35.6%	53.9%				51.5%	39.8%	63.0%	33.3%	21.0%	48.5%
Should only refer to an exercise program if within network^1^	40.0%	31.3%	49.3%				51.5%	39.8%	63.0%	21.4%	11.7%	35.9%
**FACILITATORS**												
Web/paper materials available in multiple languages	89.3%	83.8%	93.0%	91.0%	81.8%	95.8%	88.2%	78.5%	93.9%	88.1%	75.0%	94.8%
Having an exercise specialist as part of the clinical team	83.1%	76.8%	87.9%	88.1%	78.2%	93.8%	80.9%	70.0%	93.8%	78.6%	64.1%	88.3%
Referral mechanism for exercise program^2^	83.0%	76.7%	87.8%	87.9%	77.9%	93.7%	75.0%	63.6%	83.8%	88.1%	75.0%	94.8%
Patient handouts about exercise	82.5%	76.2%	87.4%	83.6%	72.9%	90.6%	77.9%	66.7%	86.2%	88.1%	75.0%	94.8%
Electronic/web-based forms with referral information	80.2%	73.7%	85.4%	79.1%	67.9%	87.1%	76.5%	65.1%	85.0%	88.1%	75.0%	94.8%
Automatic paper or electronic referral process	76.3%	69.5%	81.9%	80.6%	69.6%	88.3%	72.1%	60.4%	81.3%	76.2%	61.5%	86.5%
Posters for patients^2,3^	75.4%	68.6%	81.2%	78.8%	67.5%	86.9%	73.5%	62.0%	82.6%	73.2%	58.1%	84.3%
Practitioner education sessions about exercise*	70.6%	63.5%	76.8%	76.1%	64.7%	84.7%	57.4%	45.5%	68.4%	83.3%	69.4%	91.7%
Paper forms with referral information	67.8%	60.6%	74.2%	77.6%	66.3%	85.9%	69.1%	57.4%	78.8%	50.0%	35.5%	64.5%
Provide information to patients outside of physician visits	62.2%	54.8%	69.0%	65.7%	53.7%	75.9%	61.8%	49.9%	72.4%	57.1%	42.2%	70.9%
E-mails with written information about exercise in cancer*	58.8%	51.4%	65.8%	67.2%	55.3%	77.2%	47.1%	35.7%	58.8%	64.3%	49.2%	77.0%

Notes. *CI* Confidence Interval;

1 Participants who were exclusively exercise-related professionals did not respond to this question;

2 N=66 for Exercise-related professionals;

3 N=41 for Allied healthcare professionals;

* Indications, guidelines, referral process, safety information, etc. Agreement was measured using five-point Likert scales ranging from strongly disagree (1) to strongly agree (5). Those who somewhat agreed or strongly agreed were considered to ‘agree’ with barrier and facilitator items

## Data Availability

The datasets during and/or analyzed during the current study are available from the corresponding author on reasonable request.
