# Supplementary material for: Healthcare provider perspectives on barriers and facilitators to exercise promotion among breast cancer survivors: Differences across professions
Source: J Cancer Surviv. Author manuscript; Available in PMC 2026 Jun 5. (PMC13240992; doi:10.1007/s11764-026-02029-x)
Supplement: Supplement [file NIHMS2174537-supplement-Supplement.docx]

Supplemental Materials and Methods S1

1. **Screener Questions**
2. Have you provided care/treatment to a female breast cancer patient/survivor (i.e. a woman diagnosed with cancer) aged over 35 years in the past 12 months?
   - No^[[1]](#footnote-1)^
   - Yes
3. What is your profession? (Please select all that apply)
   - Medical oncologist
   - Radiation oncologist
   - Surgical oncologist
   - Primary care physician
   - Advanced care provider (Nurse practitioner/Physician Assistant)
   - Patient navigator/Social worker/Nurse
   - Clinical exercise physiologist
   - Cancer exercise trainer (ACSM certified)
   - Cancer exercise specialist (CETI certified)
   - Cancer exercise specialist (ACE certified)
   - Occupational therapist
   - Physical therapist
   - Other (please specify)
4. **Study Info and Consent Statement**

Consent message shown here

1. Are you still interested in participating in the study?
   - Yes
   - No^1^
2. **Professional Background**
3. How many years of professional experience do you have? Please select from the dropdown list. Range from <1 to 50 or more
4. Please describe any **formal** training/education you have had regarding physical activity and/or exercise (i.e. training/education as part of a degree). If none, please state ‘None’
5. Please describe any **continuing** training/education you have had regarding physical activity and/or exercise (e.g. training/education as part of a certification or workshop). If none, please state ‘None’
6. **Main Questions**
7. Exercise guidelines for cancer survivors endorsed by American College of Sports Medicine, American Cancer Society, etc. recommend that cancer patients work towards achieving: ≥150min/week of aerobic exercise; ≥2 days/week of muscle-strengthening exercise (resistance training). From your perspective, what percentage of breast cancer patients/survivors under your care meet exercise recommendations?
8. Clinical guidelines also reinforce the need to individualize, or personalize, exercise advice for cancer patients based on the demographic, clinical, and treatment characteristics. What percentage of breast cancer patients under your care have you offered personalized exercise advice to?
9. Do you currently have the ability to refer breast cancer patients to exercise specialists? Please select all that apply^[[2]](#footnote-2)^
   - No
   - Yes, within my healthcare network
   - Yes, outside of my healthcare network
10. The following is a list of currently published BARRIERS to discussion about exercise or referral to an exercise program. To what extent do you agree or disagree that each of the following barriers are evident in your practice? (Strongly disagree, somewhat disagree, neutral, somewhat agree, strongly agree)
    - - There is limited time during a patient visit to discuss exercise
      - I feel that there are situations in which patients that I treat where exercise would be medically unsafe
      - Patients have been told by other healthcare providers, friends, or family to rest
      - I know how to screen patients for their suitability to exercise^[[3]](#footnote-3)^3
      - I do not have the knowledge on how to where to refer a patient to exercise^2^
      - My training does not qualify me to discuss exercise or refer to an exercise program
      - I do not know how soon after surgery or treatment it is safe to start exercise
      - I should only refer to an exercise program if it is within network^2^
      - I perceive exercise to lack relevance to my patient’s cancer or symptoms
      - I am not convinced of the literature with respect to exercise and cancer outcomes
      - There are no exercise programs in my current community
      - There are limited exercise programs that understand the medical complexities of cancer survivors
    - I do not believe it is my job to discuss exercise with patients
    - My practice does not offer exercise services
    - Other (please specify)
11. The following is a list of currently published FACILITATORS of discussions about exercise or referral to an exercise program. To what extent do you agree or disagree that each of the following facilitators would be helpful in your current practice? (Strongly disagree, somewhat disagree, neutral, somewhat agree, strongly agree)
    - Patient handouts about exercise
    - Practitioner education sessions about exercise in patients with cancer (indications, guidelines, referral process, & safety information)
    - ELECTRONIC/WEB BASED forms with referral information
    - E-mails to practitioner with written information about exercise in cancer (indications, guidelines, referral process, & safety information)
    - PAPER forms with referral information
    - Having a mechanism to refer patients to an exercise program
    - AUTOMATIC paper or electronic referral process
    - Posters for patients to see so they can ask about exercise on their own accord
    - For this information to be provided to patients at a different time than at physician visits
    - Having an exercise specialist available as part of the clinical team
    - Web/paper materials available in multiple languages
    - Other (please specify)
12. **Demographics**
13. Please select your gender

- Man
- Woman

1. What is your age?
2. What state do you currently practice in?
3. How would you describe the area in which you practice?

- Urban
- Suburban
- Rural

1. What is your race and/or ethnicity? Please select all that apply

- Alaska Native
- American Indian
- Asian
- Black or African American
- Hispanic or Latino
- Middle Eastern and North African
- Native Hawaiian
- Pacific Islander
- White or Caucasian
- Other (please specify)

Supplemental Table 1. Unadjusted differences in barriers to exercise promotion among breast cancer survivors across professions

|  | **Overall** | **Exercise-related professionals** | **Oncologists / Primary care physicians** | **Allied healthcare professionals** | **Exercise-related professionals vs. Oncologists / Primary care physicians** | | **Exercise-related professionals vs. Allied healthcare professionals** | |
| --- | --- | --- | --- | --- | --- | --- | --- | --- |
|  | % | % | % | % | Φ | p | Φ | p |
| Limited time to discuss exercise | 66.7% | 53.7% | 77.9% | 69.1% | 0.255 | 0.003 | 0.152 | 0.113 |
| Patients told to rest by others | 59.7% | 59.1% | 61.8% | 57.1% | 0.027 | 0.752 | 0.019 | 0.841 |
| Limited exercise programs that understand survivor's medical complexities | 55.9% | 58.2% | 58.8% | 47.6% | 0.006 | 0.942 | 0.103 | 0.280 |
| Exercise medically unsafe for patients | 48.6% | 32.8% | 57.4% | 59.5% | 0.246 | 0.004 | 0.262 | 0.006 |
| Don't know how soon after treatment exercise is safe | 42.9% | 20.9% | 52.9% | 61.9% | 0.332 | <0.001 | 0.414 | <0.001 |
| Don't know how to screen patients for exercise suitability | 38.4% | 17.9% | 44.1% | 61.9% | 0.283 | 0.001 | 0.449 | <0.001 |
| Practice does not offer exercise services | 37.5% | 23.9% | 51.5% | 36.6% | 0.285 | <0.001 | 0.136 | 0.157 |
| Exercise lacks relevance to patient's cancer/symptoms | 33.9% | 22.4% | 57.4% | 14.3% | 0.357 | <0.001 | 0.100 | 0.297 |
| Not job to discuss exercise with patients | 29.4% | 17.9% | 47.1% | 19.1% | 0.311 | <0.001 | 0.014 | 0.881 |
| Not trained/qualified to discuss exercise or refer to an exercise program | 28.3% | 11.9% | 44.1% | 28.6% | 0.358 | <0.001 | 0.209 | 0.029 |
| No exercise programs in my current community | 27.1% | 19.4% | 44.1% | 11.9% | 0.265 | 0.002 | 0.098 | 0.305 |
| Not being convinced of the exercise and cancer outcomes literature | 25.4% | 19.4% | 39.7% | 11.9% | 0.222 | 0.010 | 0.098 | 0.305 |
| Don't know how or where to refer a patient to exercise^1^ | 44.6% | . | 51.5% | 33.3% | . | . | . | . |
| Should only refer to an exercise program if within network^1^ | 40.0% | . | 51.5% | 21.4% | . | . | . | . |

Note. CI = Confidence Interval; ^1^Participants who were exclusively exercise-related professionals did not respond to this question

Supplemental Table 2. Unadjusted differences in facilitators for exercise promotion among breast cancer survivors across professions

|  | **Overall** | **Exercise-related professionals** | **Oncologists / Primary care physicians** | | **Allied healthcare professionals** | **Exercise-related professionals vs. Oncologists / Primary care physicians** | | **Exercise-related professionals vs. Allied healthcare professionals** | |
| --- | --- | --- | --- | --- | --- | --- | --- | --- | --- |
|  | % | % | % | % | | Φ | p | Φ | p |
| Web/paper materials available in multiple languages | 89.3% | 91.0% | 88.2% | 88.1% | | 0.046 | 0.592 | 0.048 | 0.619 |
| Having an exercise specialist as part of the clinical team | 83.1% | 88.1% | 80.9% | 78.6% | | 0.099 | 0.250 | 0.127 | 0.184 |
| Referral mechanism for exercise program | 83.0% | 87.9% | 75.0% | 88.1% | | 0.165 | 0.056 | 0.003 | 0.973 |
| Patient handouts about exercise | 82.5% | 83.6% | 77.9% | 88.1% | | 0.072 | 0.406 | 0.062 | 0.517 |
| Electronic/web-based forms with referral information | 80.2% | 79.1% | 76.5% | 88.1% | | 0.032 | 0.713 | 0.115 | 0.229 |
| Automatic paper or electronic referral process | 76.3% | 80.6% | 72.1% | 76.2% | | 0.100 | 0.243 | 0.053 | 0.583 |
| Posters for patients | 75.4% | 78.8% | 73.5% | 73.2% | | 0.062 | 0.475 | 0.065 | 0.504 |
| Practitioner education sessions about exercise* | 70.6% | 76.1% | 57.4% | 83.3% | | 0.199 | 0.021 | 0.086 | 0.369 |
| Paper forms with referral information | 67.8% | 77.6% | 69.1% | 50.0% | | 0.096 | 0.264 | 0.286 | 0.003 |
| Provide information to patients outside of physician visits | 62.2% | 65.7% | 61.8% | 57.1% | | 0.041 | 0.637 | 0.086 | 0.371 |
| E-mails with written information about exercise in cancer* | 58.8% | 67.2% | 47.1% | 64.3% | | 0.203 | 0.018 | 0.030 | 0.757 |

Note. *Indications, guidelines, referral process, safety information, etc.

1. If participant selected this answer, the survey ended. [↑](#footnote-ref-1)
2. This question was only displayed to medical oncologists, radiation oncologists, surgical oncologists, primary care physicians, advanced care providers, and patient navigators/social workers/nurses. [↑](#footnote-ref-2)
3. 3 This question was included as an attention check, and reverse coded prior to analysis [↑](#footnote-ref-3)
